# Predicting Momentary Mood in Daily Life from Accelerometer Data: Evaluating Single vs. Multiple Sensor Locations Using Machine Learning

**DOI:** 10.3390/s26123688

**Published:** 2026-06-10

**Authors:** Simon Woll, Julius Müther, Dennis Birkenmaier, Gergely Biri, Ulrich W. Ebner-Priemer, Marco Giurgiu

**Affiliations:** 1 Mental mHealth Lab, Institute of Sports and Sports Science, Karlsruhe Institute of Technology, 76131 Karlsruhe, Germanyulrich.ebner-priemer@kit.edu (U.W.E.-P.); 2 Department of Embedded Systems and Sensors Engineering, FZI Research Center for Information Technology, 76131 Karlsruhe, Germany; birkenmaier@fzi.de (D.B.); biri@fzi.de (G.B.); 3 Department of Psychiatry and Psychotherapy, Central Institute of Mental Health, Medical Faculty Mannheim, Heidelberg University, 68159 Mannheim, Germany; 4 Institute of Movement Therapy and Movement-Oriented Prevention and Rehabilitation, German Sport University Cologne, 50933 Cologne, Germany; m.giurgiu@dshs-koeln.de

**Keywords:** machine learning, mood, accelerometer, mental health, wearable

## Abstract

Physical activity is a key lifestyle factor for mental health prevention, yet the influence of accelerometer placement on mood prediction remains unclear. We merged high-resolution acceleration data and Ecological Momentary Assessment (EMA) mood reports from 259 healthy participants across three ambulatory studies (SedMood, 24 hrCog, HO). Additionally, 15 min pre-assessment movement windows consisting of raw triaxial acceleration (64 Hz) from hip, thigh, chest, and wrist sensors were paired with six-item mood EMA queries. Features (e.g., mean, entropy, spectral power) were extracted and fed into gradient-boosted decision tree models (XGBoost), trained separately for energetic arousal, valence, and calmness. Performance was measured using the metrics MAE, RMSE and R^2^. Within individual studies, chest and hip sensors achieved the highest performance, followed by wrist and thigh. In the combined dataset, hip sensors again outperformed thigh (R^2^ 0.38 vs. 0.20). Multi-sensor models rarely surpassed the best single-sensor configuration and sometimes reduced accuracy. These results suggest that sensor location modestly impacts mood-prediction performance, with hip and chest offering the most reliable signals, while adding sensors does not reliably enhance predictive power. Future work should explore larger, homogenous datasets and location-specific feature engineering to refine wearable-based mental health monitoring.

## 1. Introduction

Physical activity can significantly improve overall well-being and, beyond that, help prevent physical and mental health disorders. For example, research shows that physical activity can reduce symptoms of depression and anxiety [1,2,3], improve mood [4], and enhance productivity [5]. Therefore, physical activity in daily life (e.g., walking in the garden, taking the stairs) [6] as well as structured exercise (e.g., jogging sessions) [7], alongside other factors such as sleep behavior, diet and nutrition, or social connection, constitute relevant lifestyle factors that are particularly important for both the prevention and intervention of mental health issues.

To gain insight into high-resolution movement data in everyday life, wearables have become increasingly important tools in recent years. These devices record raw acceleration data to derive indicators such as energy expenditure, intensity levels (e.g., sedentary to vigorous), posture (e.g., sitting vs. standing), and activity types (e.g., walking, cycling) [8]. Outside of research contexts, wearable devices have become a leading fitness trend with a growing consumer market [9,10,11]. They are cost-effective, easy to use, and capable of continuously monitoring activity while providing feedback on physical activity, sleep, and other physiological signals.

The body location where the accelerometer is placed has a major influence on the accuracy of the measured values. For example, if total body movement is to be measured, it is best to place the device close to the body’s center of gravity (e.g., hip or thigh) [12]. If body postures are to be measured, such as distinguishing between sitting/lying and standing, the thigh location is preferred for the accelerometer [13]. To record detailed movements of the extremities, placement at the wrist or ankle is preferable [14].

Combining sensor data with self-report methods like Ecological Momentary Assessment (EMA) provides a comprehensive dataset to gain insight into mental health [15]. The term mental health encompasses a broad spectrum of psychological constructs, including emotional well-being, affective states, and mood. Among these, mood stands out as a core indicator of psychological health [16]. It changes subtly throughout the day and exerts a continuous influence on perception, thinking, and behavior [17]. The assessment of mood is well established in EMA research and can be conducted using items developed by Wilhelm and Schoebi [18]. These data are essential for mental health research, as they help analyze the influence of activity patterns, sleep behavior, and other relevant markers on mental states and traits. For example, research findings indicate that even brief episodes of movement can improve affective well-being [6]; sleep disturbances are linked to various psychiatric conditions [19]; and higher daily step counts are associated with fewer depressive symptoms [20].

The high-frequency data collected through wearables and additional self-reports via EMA create large, complex datasets that often exceed the capabilities of conventional analysis methods [21]. Basically, most existing analyses relating physical activity to mood are based on highly aggregated data; i.e., 64 Hz of three-dimensional acceleration is aggregated across 60 s into a single value (instead of 11,520 values). Emerging technologies, especially artificial intelligence and machine learning (ML), open new possibilities. ML algorithms can identify hidden patterns, model complex interdependencies, and generate real-time predictions from high-dimensional data. A systematic literature review by Timm et al. identified 66 ambulatory assessment studies examining the relationship between physical activity and affective well-being. Most studies primarily investigated incidental and unstructured physical activity (e.g., climbing stairs) and found positive associations with affective well-being. Overall, the findings were heterogeneous, and the review raises the question of potential sources for this heterogeneity (e.g., assessment methods, design features, or statistical methods). For instance, these studies employed various sensor locations, including the hip (30 studies), wrist (18), chest (11), and thigh (eight), with seven studies incorporating multiple wearing locations. Notably, none of these studies applied ML methods for data analysis, and among those using multiple sensors, no systematic comparison of sensor locations or multi-sensor configurations was conducted. Similarly, a more recent review by Woll et al., which focused on studies integrating wearable-based physical activity data and ML techniques to infer mental health states and traits, identified 49 studies [22]. Again, none of these studies investigated the role of sensor location, and none integrated multiple accelerometer sensors at different wearing locations. These gaps highlight a critical need for research examining the influence of sensor location, particularly in the context of mood prediction based on acceleration signals via ML models.

Based on three studies that are largely consistent in their methodology, this study investigates whether the sensor wearing location influences the predictive capability of ML models when predicting mood from raw accelerometer data. In detail, our first analysis investigates how predictive capabilities are influenced by the wearing location (hip vs. thigh vs. chest vs. wrist) within each study and with combined datasets across all studies. Additionally, in a secondary analysis, we investigate whether multi-sensor systems combining accelerometers at diverse wearing locations can positively influence predictions.

## 2. Materials and Methods

In total, data from 259 healthy participants from three separate ambulatory assessment studies conducted between 2019 and 2023 were combined. Of the participants, 58.7% were female; the mean age was 30.07 ± 10.04 years (range: 18–62 years), and the mean body mass index (BMI) was 22.88 ± 3.04 kg/m^2^. Eligibility criteria required all participants to be physically capable of performing daily activities without restrictions (i.e., free from injury or chronic illness). Each study received prior approval from the appropriate local ethics committee. Participants provided written informed consent and were free to withdraw from the studies at any time.

All studies maintained methodological consistency in data collection: physical activity was assessed using move3 and move4 accelerometers (movisens GmbH, Karlsruhe, Germany), and mood was assessed via electronic diaries utilizing smartphones and the movisensXS app (version 0.7.47574). The individual studies varied in their specific objectives and target populations. The SedMood study focused on the association between sedentary behavior and mood among 92 university employees over five days. Sedentary behavior was captured via multiple sensors placed on the hip, chest, and thigh. Mood data were collected up to ten times daily. For more information, see Giurgiu et al. (2019) [23]. The 24hrCog study aimed to investigate within-person associations between physical behavior and self-perceived work ability; this study involved 110 university students. Participants wore accelerometers on the wrist, hip, and thigh continuously over five days. Mood was recorded up to six times per day. Details are reported in Giurgiu et al. (2021) [5]. The HO study examined physical behavior in different work settings (home vs. office) across five days in 57 working adults. Participants wore accelerometers on the thigh and hip for continuous 24 h monitoring. Mood was assessed up to seven times per day. See Sers et al. (2023) for further details [24].

Momentary mood was assessed over time using a short scale consisting of six bipolar items developed and validated by Wilhelm and Schoebi [18]. These six bipolar items were presented in mixed order, with three items in reversed polarity, on visual analog scales ranging from 0 to 100. Items included: *EA*1—tired to awake; *V*1—content to discontent; *C*1—agitated to calm; *EA*2—full energy to without energy; *V*2—unwell to well; *C*2—relaxed to tense. These items were combined to form the three basic mood dimensions: energetic arousal (*EA*), valence (*V*) and calmness (*C*), equivalent to the description by Wilhelm and Schoebi:

(1) $$EA=\frac{EA1+(100-EA2)}{2}V=\frac{V1+(100-V2)}{2}C=\frac{C1+(100-C2)}{2}$$

Physical activity was assessed using move3 (SedMood) and move4 (24hrCog, HO) sensors. Despite differences in device generations, the mechanical design and output format remained consistent. Devices recorded triaxial acceleration within ±16 g at 64 Hz. Raw accelerometer data were extracted using movisens SensorManager (version: 1.16.4) and used directly for analysis without preprocessing or parameterization (e.g., no filtering or conversion to energy expenditure or step count). To compare the predictive potential of each location—as well as combinations of multiple placements—we utilized all available sensor wearing locations in our dataset: hip, thigh, chest, and wrist.

Although mood is a complex construct that cannot be explained by physical activity alone, in order to minimize external influences and focus exclusively on the wearing location of the device, we have used physical activity as the sole input variable for our models—even though this carries the risk of compromising performance.

A Python-based machine learning framework was implemented to handle data extraction and preprocessing, model training, validation, and testing. Implementation was done using Python 3.11. All models were trained on HAICORE (https://www.nhr.kit.edu/userdocs/haicore/hardware/, accessed on 2 August 2025) using NVIDIA A100-40 GPUs. Our models were implemented using XGB, a gradient-boosted decision tree model known for its scalability and strong performance across tasks, especially in mental health prediction using structured data [22]. While it does not model temporal sequences directly, it can capture time-based patterns through our engineered features.

The datasets were cleaned by removing participants with completely missing mood or acceleration data; additionally, we removed mood samples with no corresponding acceleration data. For all studies, custom extractors were implemented to standardize extracted dataset formats. Mood values for the three mood dimensions (EA, V, and C) were extracted. In line with previous studies using 15 min of preceding physical activity to analyze the association of physical behavior to valence, energetic arousal, and calmness [6], for each mood assessment, 15 min of preceding raw accelerometer data were used as the corresponding movement input. Features were extracted from these segments using tsfel (v0.1.9) and included: mean, standard deviation, maximum, minimum, entropy, skewness, kurtosis, absolute energy, maximum power spectrum, fundamental frequency, neighborhood peaks, zero-crossing rate, and autocorrelation. The initial feature set comprised over 400 descriptors covering three main domains: temporal, statistical, and spectral. To analyze the feature set, we utilized the integrated feature-importance analysis functions of XGB [25]. Statistical features quantified the overall distribution, variability, and energy of the activity signal within each segment. Spectral features captured the dominant periodic components and frequency-domain energy distribution. Temporal features characterized short-term dynamics, rhythmicity, and self-similarity over time. Together, these features provided a compact yet expressive representation of both the signal’s amplitude distribution and its temporal structure, enabling the models to capture temporal patterns indirectly through fixed-length segment descriptors. The final set of 13 features was verified using simple feature-ablation models that further reduced the initial feature pool; although 2 models showed modest performance gains, most models performed substantially worse, so we retained the 13-feature set for all models to ensure consistent comparisons. Feature standardization was done using a standard scaler by removing the mean and scaling to unit variance. Libraries used included pyunisens (v1.5.0) and pandas (v2.2.0).

For the multi-sensor and multi-study analyses, we utilized a data pooling approach rather than feature concatenation. Specifically, instead of increasing the input feature vector length by concatenating data from different sensors, we increased the total number of training samples by treating each sensor’s 15 min segment as an independent observation linked to the same mood rating. This ensured that the model input dimension remained constant across all analyses while allowing the model to learn from a more diverse and larger set of movement patterns.

After data extraction and preprocessing, we trained each model on the respective dataset to predict the EA, V, and C dimensions independently. To ensure robust performance estimates, we performed 5-fold cross-validation for each model. Folds were constructed on the participant level, meaning all samples from one participant appear only in one fold. Performance was evaluated using mean absolute error (MAE), which describes the mean difference between predicted and actual values; thus, an MAE value of five indicates that, on average, predictions deviate by a maximum of five points from the actual value. A lower MAE value indicates better performance, with zero representing optimal predictive capability. Additionally, we report the root mean squared error (RMSE), which is similar to the MAE but more sensitive to outliers, and the R^2^ values, which compare the model’s performance to a mean baseline predictor (a model that always predicts the mean value of the dataset). We calculated performance values for every fold individually and report mean ± standard deviation for all MAE, RMSE and R^2^ values. Model training and evaluation were performed using scikit-learn (v1.6.1) and XGBoost (v2.1.3). For statistical comparison of measured MAE values between sensor locations, paired t-tests were conducted using the participant-wise MAE values obtained for each sensor location.

## 3. Results

### 3.1. Dataset

Data were collected from a total of 259 participants across three studies, encompassing 1384 days of concurrent mood and movement recordings and resulting in 8071 fully completed mood questionnaires. This corresponds to a mean of 5.34 days of data collection and 31.16 mood assessments per participant. An overview of the collected data for each study is presented in Table 1.

Four distinct sensor wearing locations were employed across the studies: hip, thigh, chest, and wrist. While the hip and thigh sensors were utilized in all three studies (N = 259), the SedMood study included an additional sensor at the chest (N = 92), and the 24hrCog study added a wrist sensor (N = 110).

### 3.2. In-Study Comparison of Different Wearable Locations

The first analysis aimed to evaluate how prediction accuracy varied by wearing location (thigh, hip, chest, wrist) within each study (SedMood, 24hrCog, HO). Performance values are reported in Table 2.

When comparing the hip and thigh sensor locations, which were available in all three studies, the hip location outperformed the thigh location in every instance. In the SedMood study, an additional chest-worn sensor was included, which outperformed both the hip- and thigh-based models of the SedMood study. In the 24hrCog study, additional acceleration data recorded at the wrist showed better performance than the thigh position but lower performance than the hip position of the same study.

Across the three studies, paired-sample t-tests showed that sensor location significantly affected MAE, although the pattern differed by study. In Study 1, the hip yielded a significantly lower MAE than the thigh across all outcomes, with mean differences ranging from −3.52 to −4.57, t(107) = −15.72 to −18.54, all *p* < 0.001, and large effects (d = −1.51 to −1.78); the hip also outperformed the wrist with mean differences from −1.58 to −2.10, t(92) = −6.24 to −8.40, all *p* < 0.001, and medium-to-large effects (d = −0.65 to −0.87). In the same study, the wrist showed a significantly lower MAE than the thigh, with thigh–wrist mean differences of 2.09 to 2.51, t(93) = 7.52 to 10.03, all *p* < 0.001, and medium-to-large to large effects (d = 0.78 to 1.03). In Study 2, thigh and hip MAE did not differ significantly for EA, M diff = 0.11, t(51) = 0.43, *p* = 0.334, d = 0.06, whereas the hip showed a significantly lower MAE for V, M diff = 0.86, t(51) = 3.03, *p* = 0.002, d = 0.42, and C, M diff = 1.75, t(51) = 4.86, *p* < 0.001, d = 0.67. In Study 3, thigh–hip differences were small and not robust for EA and V, M diff =−0.03 and −0.11, *p* = 0.443 and 0.273, respectively, and small for C, M diff = 0.37, t(89) = 1.94, *p* = 0.028, d = 0.20, while the chest showed a significantly lower MAE than both hip and thigh across all outcomes, with hip–chest mean differences of 2.97 to 3.43 and thigh–chest mean differences of 2.62 to 3.31, all *p* < 0.001, and large effects (d = 1.18 to 1.79).

### 3.3. Between-Study Comparison of Different Wearable Locations

To maximize the available training data samples for each sensor location, we aggregated the three studies into a single comprehensive dataset per sensor location. The aim was to evaluate the consistency and generalizability of model performance across diverse study conditions when varying the sensor wearing location. However, as not all wearable locations were present in every study, the resulting dataset sizes varied by sensor location. Consequently, comparisons involving wrist and chest sensors are less heterogeneous than those involving hip and thigh sensors, due to substantially smaller sample sizes and the fact that only one study employed the respective location (chest employed in the SedMood study and wrist employed in the 24hrCog study). Thus, this analysis focuses solely on the hip and thigh wearing locations. For each sensor location, we utilized the complete available dataset and trained models specifically tailored to that location. Table 3 provides performance measurements of the models per wearable location.

Similar to the previous analysis at the study level, models based on hip data delivered better performance values than thigh-based models. When comparing the performance of the combined dataset models to the single-study dataset models, we observed a slight to moderate decrease in performance.

### 3.4. In-Study Combination of Wearable Locations

To combine the advantages of different wearing locations, sensor data from all available locations within each study were pooled in a single training data set per study to evaluate model performance when the model is trained on diverse body placements simultaneously. Specifically, we merged thigh, hip, and chest data for the SedMood study; thigh, hip, and wrist data for the 24hrCog study; and thigh and hip data for the HO study. This approach allows for the assessment of algorithm robustness under conditions of sensor variability. Additionally, to facilitate better between-study comparison, we included the hip/thigh combination for the SedMood and 24hrCog studies as well. Performance results for each study are shown in Table 4.

When pooling data from diverse wearable locations into a joint dataset per study, we observed overall lower performance compared to the best models, whose training data originated from a single sensor location, in most cases. The HO study was an exception, where performance values were similar when comparing the pooled model to models trained on purely thigh or hip sensor data. In the SedMood study, the pooled hip/thigh model outperformed the thigh-only model but showed lower predictive accuracy than the hip-only model. Crucially, while adding chest samples to the pool improved performance relative to the hip/thigh model, the results remained slightly worse compared to the model trained exclusively on chest data. In the 24hrCog study, pooling hip and thigh samples resulted in lower performance than using either location individually. The inclusion of wrist data into training dataset led to a further decrease in predictive accuracy.

### 3.5. Between-Study Combination of All Wearable Locations

The final analysis assessed model performance on the combined dataset from all three studies, incorporating all available sensor locations (thigh, hip, chest, wrist). This configuration represents the most heterogeneous dataset across all analyses and evaluates the models’ ability to generalize across diverse populations, study contexts, and sensor setups. The corresponding performance values are presented in Table 5.

Combining all wearable locations from all three studies into a single dataset led to slightly better performance compared to the combined model of the 24hrCog study, but lower performance compared to the SedMood and HO studies.

### 3.6. Baseline Models

To improve interpretability, we calculated mean baseline models for every dataset. The baseline models always predict the mean values, which results in R^2^ values of 0. In Table 6 the performance values of the baseline models for the three studies and the pooled dataset across all study datasets are shown.

## 4. Discussion

The aim of this study was to examine whether sensor locations influence the performance of machine learning models in predicting momentary mood from raw accelerometer data. Additionally, we investigated whether combining multiple sensor locations enhances model performance. The results indicate that: (i) among the analyzed wearing positions, in the SedMood study, where chest data were available, the chest performed best, followed by the hip, which outperformed the thigh in all studies. In the 24hrCog study, which incorporated an additional wrist sensor, the wrist exhibited the poorest performance compared with the other models of the 24hrCog study; and (ii) multi-sensor configurations (i.e., combining data from multiple wearing locations) did not outperform single-sensor setups and, in some cases, even reduced model performance.

When comparing single-sensor models, the results indicate that sensors positioned near the center of mass (e.g., hip or chest) yield higher predictive performance than sensors placed on the extremities (e.g., thigh or wrist). Combining the three study datasets increased the sample size but reduced performance relative to the single-study datasets, likely due to increased heterogeneity. When directly comparing the most common hip and thigh placements, the differences were small: in the combined dataset, the maximum advantage was 0.18 R^2^ (or 2.29 MAE), and in the single 24hrCog study dataset, the maximum was 0.24 R^2^ (or 4.69 MAE). These results indicate that sensor location yields observable, albeit modest, differences in predictive performance. Among the analyzed positions, the hip consistently showed a marginal advantage over the thigh across all studies. While the absolute differences in error metrics were relatively small, the directional consistency of this finding across three independent datasets suggests that the hip may be a more robust location for capturing mood-relevant movement patterns. However, the narrow performance margins also imply that for many practical applications, the choice of location may be guided as much by participant comfort and compliance as by predictive gain.

To our knowledge, no prior work has compared wearing locations for momentary mood prediction from acceleration data, although Cleland et al. [26] reported a marginal effect of accelerometer location on activity-recognition performance. Given the modest benefit of the hip over the thigh and wrist, the hip is recommended for field applications of mood prediction. The chest, which performed best in the SedMood study, is also viable; however, only the SedMood dataset included both chest and hip placements in which the chest compared favorably.

Regarding multi-sensor pooling, the models trained on combined samples from multiple locations did not outperform single-location models. Thus, integrating multiple accelerometer locations into a unified dataset does not appear to yield a clear improvement in model performance. Similar to the single wearing location comparison, Cleland et al. [26] analyzed the use of multiple accelerometers for the detection of everyday activities and found only a slight advantage when using multiple locations, with a decreasing effect when three or more sensors were added. The use of two or three sensors placed on distinct body regions (e.g., upper vs. lower body or extremities) may enrich the training distribution by capturing complementary movement patterns, potentially enhancing model performance. However, the inclusion of samples from locations with lower predictive power (e.g., thigh) alongside higher-performing locations (e.g., hip) may introduce noise or less discriminative patterns into the training set. If the relationship between movement and mood is location-specific, pooling these distinct signals into a single model may lead to a “regression to the mean” effect, where the model fails to capture the optimal patterns unique to the best-performing locations. It is therefore plausible that while additional sensors may enhance the granularity of movement detection, the resulting improvement in model performance is likely to be modest. Moreover, there is a risk that certain signals become overrepresented in the model, potentially leading to biased predictions. Future work could exploit location-specific features (e.g., wrist data for hand movements, chest or hip for intensity, thigh for posture) to enhance model performance.

A key consideration in our multi-sensor analysis was the choice of a data pooling approach over multimodal feature fusion. By treating each sensor segment as an independent observation, we maintained a constant input dimensionality, allowing for a direct comparison between single-sensor and multi-sensor configurations without the con-founding effect of changing model complexity. However, this approach does not explicitly model the spatial correlation between different body parts. Nevertheless, given the heterogeneity of our datasets, where not all participants wore the same sensor combinations, pooling provided a robust way to leverage all available data without the complexity of handling the high-dimensional, sparse feature matrices that arise when sensors are missing.

Despite the methodological homogeneity of the studies (i.e., using raw accelerometry signals and consistent momentary mood assessments), the use of three independent datasets posed several challenges. A comparison of model performance revealed that some study datasets performed better than others. These differences can be attributed to the heterogeneous nature of the studies, each conducted with different aims and populations. While the SedMood study specifically targeted the relationship between physical behavior and mood, the 24hrCog study focused on associations between physical activity and self-perceived work ability, and the HO study examined physical behavior across different occupational settings. Moreover, the SedMood dataset featured the largest number of samples (3754), compared to 2621 in the 24hrCog study and 1696 in the HO study. It also had the highest average number of mood assessments per participant per day (8.0), compared to 4.8 (24hrCog) and 4.9 (HO), which may further contribute to its superior model performance.

To comprehensively understand a complex construct such as mood, raw acceleration data alone is likely insufficient. Additional physiological signals, such as heart rate or ECG, can further inform models but might require specific sensor locations. For instance, ECG signals are best captured at the chest, while heart rate is reliably measured at the wrist. Enriching passively acquired sensing data with other influential contextual information—such as environmental influences [27] or weather [28]—can enhance predictive accuracy. However, such contextual data (e.g., weather) cannot be derived from sensors alone and require the integration of external data sources.

Methodologically, our approach relied on extracting aggregated descriptors from 15 min windows to represent movement. While these features capture essential signal characteristics, they do not model the raw temporal sequences directly. In contrast, deep learning architectures, such as Convolutional Neural Networks (CNNs) or Recurrent Neural Networks (RNNs), could potentially extract more nuanced temporal dynamics. However, such sequence-based models often require significantly larger datasets to outperform gradient-boosted trees like XGB and may offer less interpretability. Future studies should investigate whether the automated feature learning of deep models provides a meaningful advantage over traditional feature engineering for mood prediction, particularly as larger multi-sensor datasets become available.

To enable long-term monitoring with minimal burden in everyday life, sensor placement should be optimized to allow for a wide range of measurements using as few devices as possible. In this context, wearable devices such as smartwatches (wrist) or smartphones (e.g., carried in a pocket) offer practical solutions, as several studies have recorded an increase in compliance when switching to wrist sensors [13,29,30]. Future research endeavors should aim to integrate multimodal data to capture the multifactorial nature of mood and support scalable, unobtrusive mental health monitoring. Furthermore, recent machine learning approaches—especially deep learning techniques—enable multimodal sensor fusion or domain adaptation, which can substantially enhance a model’s predictive performance.

Our study has several limitations. First, our dataset is relatively small for ML applications, which may contribute to the mixed results overall. However, we are not aware of any study that has combined acceleration signals and momentary mood ratings in over 250 participants [22]. Second, our dataset is inhomogeneous in terms of different study characteristics and was not collected for the specific purpose of this analysis. While this is generally beneficial for generalizability, a more homogeneous dataset would have been helpful to minimize external influences when analyzing the effects of sensor positioning and comparing single- and multi-sensor models. Third, no dataset included all four wearing locations, making a comprehensive overall comparison impossible. Moreover, in our study, the number of data points from wrist and chest locations was underrepresented compared to hip and thigh locations. Therefore, replication with larger datasets may be required. Fourth, our results show that physical activity alone does not sufficiently explain mood variations, indicating that additional signals are required to obtain statistically significant predictions from passively collected data—an essential prerequisite for clinical or real-world applications.

## 5. Conclusions

Wearing location is a central consideration in the design of wearable-based study protocols and is often tailored to the specific research objective. For instance, the wrist is commonly used for sleep applications [31], while the thigh location is preferred for accurately differentiating between standing and sitting/lying postures [13]. Sensor placement also varies in studies investigating the association between mood and physical activity [6,22], although its specific influence on predictive accuracy has not been systematically evaluated. As the first study to address this question using advanced ML techniques, our findings suggest that while the impact of wearing location on mood prediction from accelerometer data is small, sensors located at the hip or chest yielded consistently better results compared to the thigh or wrist across the different study contexts. Moreover, the integration of multiple sensor locations does not appear to substantially improve model performance. In longitudinal investigations where measurements are taken over weeks, months, or years, the placement of sensors plays a decisive role. To ensure high compliance and comfort, researchers should prioritize locations that impose the least possible burden on participants. At the same time, the chosen placement must be justified by the study’s primary aims, guaranteeing that the collected data remain both valid and relevant. To further strengthen evidence-based recommendations for optimal sensor placement, larger high-quality EMA datasets are needed—ideally with raw data access, dense sampling, and heterogeneous samples.

## Figures and Tables

**Table 1:** Overview of datasets by study.

Study	N	Days Per Person (⌀)	Mood Assessments Per Day Per Person (⌀)	Σ Completed Mood Questionnaires	Σ Days with Mood Assessments	Wearable (Locations)
SedMood	92	5.1	8.0	3754	465	move3 (thigh, hip) move3-ecg (chest)
24hrCog	110	5.0	4.8	2621	553	move4 (thigh, hip, wrist)
HO	57	6.1	4.9	1696	366	move4 (thigh, hip)
SUM	259			8071	1384	

**Table 2:** Performance by study and sensor location. Values are reported as mean ± standard deviation.

			R^2^			MAE			RMSE	
Study	Location	EA	V	C	EA	V	C	EA	V	C
SedMood Study	thigh	0.28 ± 0.07	0.16 ± 0.10	0.26 ± 0.08	14.87 ± 0.90	11.96 ± 0.90	13.81 ± 0.90	18.47 ± 0.91	14.97 ± 0.91	17.20 ± 0.92
	hip	0.37 ± 0.07	0.25 ± 0.09	0.38 ± 0.06	13.76 ± 0.83	11.21 ± 0.72	12.58 ± 0.82	17.30 ± 0.83	14.14 ± 0.86	15.84 ± 0.86
	**chest** *	**0.42 ± 0.10**	**0.35 ± 0.11**	**0.44 ± 0.08**	**12.96 ± 1.17**	**10.34 ± 1.13**	**11.76 ± 0.94**	**16.62 ± 1.38**	**13.16 ± 1.17**	**14.98 ± 1.04**
24hrCog Study	thigh	0.43 ± 0.03	0.30 ± 0.05	0.44 ± 0.03	14.46 ± 0.46	11.97 ± 0.63	11.95 ± 0.48	17.89 ± 0.54	15.25 ± 0.50	15.16 ± 0.42
	**hip**	**0.67 ± 0.05**	**0.62 ± 0.05**	**0.66 ± 0.06**	**9.77 ± 0.72**	**7.90 ± 0.86**	**8.38 ± 0.76**	**13.62 ± 0.93**	**11.25 ± 0.80**	**11.85 ± 0.98**
	wrist	0.58 ± 0.06	0.52 ± 0.07	0.58 ± 0.06	11.82 ± 1.64	9.45 ± 1.56	9.94 ± 1.58	15.32 ± 1.12	12.59 ± 0.85	13.13 ± 1.00
HO Study	thigh	0.54 ± 0.06	0.55 ± 0.07	0.52 ± 0.08	12.69 ± 1.15	10.63 ± 1.11	11.82 ± 1.30	15.88 ± 1.11	13.90 ± 1.05	15.34 ± 1.24
	**hip**	**0.57 ± 0.04**	**0.61 ± 0.05**	**0.64 ± 0.04**	**12.12 ± 0.76**	**9.62 ± 0.79**	**9.93 ± 0.76**	**15.25 ± 0.76**	**12.97 ± 0.79**	**13.31 ± 0.76**

* The bold values mark the overall best performance per study. MAE = mean absolute error, RMSE = root mean squared error, EA = energetic arousal, V = valence, C = calmness.

**Table 3:** Performance values for hip and thigh locations across combined datasets. Values are reported as mean ± standard deviation.

		R^2^			MAE			RMSE	
Location	EA	V	C	EA	V	C	EA	V	C
thigh	0.20 ± 0.07	0.25 ± 0.08	0.23 ± 0.07	16.66 ± 0.83	13.01 ± 0.84	14.50 ± 0.82	20.38 ± 0.85	16.45 ± 0.85	18.11 ± 0.83
**hip** *	**0.38 ± 0.06**	**0.36 ± 0.07**	**0.35 ± 0.06**	**14.37 ± 0.87**	**11.83 ± 0.84**	**13.14 ± 0.78**	**17.94 ± 0.88**	**15.16 ± 0.83**	**16.69 ± 0.79**

* The bold values mark the overall best performance per study. MAE = mean absolute error, RMSE = root mean squared error, EA = energetic arousal, V = valence, C = calmness.

**Table 4:** Performance results using all sensor locations per study. Values are reported as mean ± standard deviation.

		R^2^			MAE			RMSE	
Study (Locations)	EA	V	C	EA	V	C	EA	V	C
SedMood Study (hip, thigh, chest)	0.38 ± 0.10	0.24 ± 0.15	0.36 ± 0.11	13.80 ± 1.38	10.99 ± 1.36	12.72 ± 1.36	17.23 ± 1.39	14.20 ± 1.36	16.10 ± 1.36
SedMood Study (hip, thigh)	0.33 ± 0.06	0.21 ± 0.09	0.31 ± 0.07	14.37 ± 0.82	11.33 ± 0.81	13.25 ± 0.81	17.92 ± 0.81	14.45 ± 0.81	16.65 ± 0.82
24hrCog Study (hip, thigh, wrist)	0.25 ± 0.08	0.09 ± 0.09	0.23 ± 0.09	16.85 ± 0.75	13.72 ± 1.25	14.31 ± 1.13	20.52 ± 1.03	17.32 ± 0.94	17.82 ± 1.07
24hrCog Study (hip, thigh)	0.40 ± 0.07	0.29 ± 0.10	0.34 ± 0.08	14.91 ± 1.01	11.87 ± 1.05	12.98 ± 0.99	18.39 ± 1.05	15.32 ± 1.06	16.45 ± 1.00
HO Study (hip, thigh)	0.59 ± 0.05	0.61 ± 0.06	0.61 ± 0.05	11.89 ± 0.93	9.65 ± 0.97	10.32 ± 0.97	15.04 ± 0.93	12.88 ± 0.99	13.74 ± 0.93

MAE = mean absolute error, RMSE = root mean squared error, EA = energetic arousal, V = valence, C = calmness.

**Table 5:** Performance values for the aggregated dataset of all studies using all sensor locations. Values are reported as mean ± standard deviation.

	R^2^			MAE			RMSE	
EA	V	C	EA	V	C	EA	V	C
0.28 ± 0.09	0.29 ± 0.11	0.25 ± 0.10	15.72 ± 1.06	12.54 ± 1.04	14.29 ± 1.04	19.30 ± 1.27	16.02 ± 1.24	17.89 ± 1.18

MAE = mean absolute error, RMSE = root mean squared error, EA = energetic arousal, V = valence, C = calmness.

**Table 6:** Simple mean baseline model performance values for every dataset.

		R^2^			MAE			RMSE	
Dataset	EA	V	C	EA	V	C	EA	V	C
SedMood Study	0	0	0	21.82	16.30	20.06	17.96	12.84	16.03
24hrCog Study	0	0	0	23.72	18.17	20.29	19.94	14.81	15.85
HO Study	0	0	0	23.37	20.68	22.07	20.34	16.54	17.88
Pooled Dataset	0	0	0	22.81	18.96	20.66	19.02	15.07	16.92

MAE = mean absolute error, RMSE = root mean squared error, EA = energetic arousal, V = valence, C = calmness.

## Data Availability

The datasets used in our study were combined from different studies. Data from the individual studies are either publicly available or must be requested from the owner who conducted the respective study. The code developed for this study is publicly available on GitHub and can be found here: https://github.com/fzi-forschungszentrum-informatik/beactive (accessed 28 November 2025). Model development and testing were done using Python v3.11 and publicly available packages (including scikit-learn, pandas, numpy, XGBoost, tsfel, pyunisens).

## Abbreviations

The following abbreviations are used in this manuscript:

EMA	Ecological Momentary Assessment
ML	Machine Learning
EA	Energetic Arousal
V	Valence
C	Calmness
MAE	Mean Absolute Error
RMSE	Root Mean Squared Error
